# A Comparative Study of ECG-derived Respiration in Ambulatory Monitoring using the Single-lead ECG

**DOI:** 10.1038/s41598-020-62624-5

**Published:** 2020-03-31

**Authors:** Carolina Varon, John Morales, Jesús Lázaro, Michele Orini, Margot Deviaene, Spyridon Kontaxis, Dries Testelmans, Bertien Buyse, Pascal Borzée, Leif Sörnmo, Pablo Laguna, Eduardo Gil, Raquel Bailón

**Affiliations:** 1 Delft University of Technology, Circuits and Systems (CAS) group, Delft, 2600 AA the Netherlands; 2 KU Leuven, Department of Electrical Engineering-ESAT, STADIUS Center for Dynamical Systems, Signal Processing and Data Analytics, Leuven, 3001 Belgium; 3 University of Connecticut, Department of Electrical Engineering, Storrs, CT 06268 USA; 4 University of Zaragoza, BSICoS Group, Aragón Institute of Engineering Research (I3A), IISAragon, Zaragoza, 50015 Spain; 5 CIBER de Bioingeniería, Biomateriales y Nanomedicina (CIBER-BBN), Madrid, Spain; 6 University College London, Institute of Cardiovascular Science, London, WC1E 6BT UK; 7 University College London, Barts Heart centre at St Bartholomews Hospital, London, EC1A 7BE UK; 8 UZ Leuven, Department of Pneumology, Leuven, 3001 Belgium; 9 Lund University, Department of Biomedical Engineering, Lund, 118, 221 00 Sweden

**Keywords:** Cardiology, Health care

## Abstract

Cardiorespiratory monitoring is crucial for the diagnosis and management of multiple conditions such as stress and sleep disorders. Therefore, the development of ambulatory systems providing continuous, comfortable, and inexpensive means for monitoring represents an important research topic. Several techniques have been proposed in the literature to derive respiratory information from the ECG signal. Ten methods to compute single-lead ECG-derived respiration (EDR) were compared under multiple conditions, including different recording systems, baseline wander, normal and abnormal breathing patterns, changes in breathing rate, noise, and artifacts. Respiratory rates, wave morphology, and cardiorespiratory information were derived from the ECG and compared to those extracted from a reference respiratory signal. Three datasets were considered for analysis, involving a total 59 482 one-min, single-lead ECG segments recorded from 156 subjects. The results indicate that the methods based on QRS slopes outperform the other methods. This result is particularly interesting since simplicity is crucial for the development of ECG-based ambulatory systems.

## Introduction

Continuous monitoring of respiration plays a key role in the detection and management of different conditions, such as stress^1,2^ and sleep disorders^3,4^. Biomarkers like respiratory rate, breathing phases, and tidal volume are relevant for the detection of mental stress^1^, anxiety^2^, and sleep apnea events^5,6^. In addition, the coupling between respiration and heart rate has been used as a biomarker for the aforementioned conditions^7,8^ as well as for the understanding of the interactions between the cardiac and respiratory systems^9^.

Despite the importance of monitoring respiration, its recording requires the use of invasive and intrusive sensors like thermistors, spirometers, and respiratory belts. Even though these sensors are regularly used, for instance during polysomnographic recordings, their use in ambulatory systems is very limited since they not only interfere with natural breathing, but are often associated with high costs and low comfort. Different studies have shown that the respiratory rate, and even the respiratory wave morphology, can be approximated by ECG-derived respiration (EDR)^5,10–20^. The derived signal is defined by certain morphological properties of the ECG particularly influenced by respiration. This influence can be explained by the respiratory-induced chest movements that cause changes in the position of the electrodes relative to the cardiac vector^21^. Moreover, the filling and emptying of the lungs cause changes in the electrical impedance of the chest. As a result, the morphology of the ECG is modulated by respiration.

Different methods to compute the EDR have been proposed in the literature. Here, methods based on the single-lead ECG will be investigated, as such methods are commonly used in ambulatory monitoring systems. One type of method tracks changes in either R- and S-wave amplitude^10^ or the difference in R-S amplitude^22^. Changes in R-wave amplitude have been used for the detection of sleep apnea^6^. Another method explores the area of the QRS complexes^5,10^, also called R-wave area^23^, which has been used for detection of sleep apnea^24,25^ and for extraction of respiratory information from conductive textile electrodes^26^. Yet another type of method extracts the EDR from changes in the slopes and angles of the QRS complexes^13,27^, having been evaluated for respiratory rate estimation under noisy and nonstationary conditions, but not for wave morphology approximation. In^14^, the EDR was defined by the largest decrease of the 4th central moment of the ECG in segments between R- and S-waves; this method was evaluated for respiratory rate estimation only^28^. More advanced methods extract morphological information from the whole QRS complexes using either principal component analysis (PCA)^11^ or its kernel version^12^. Other decomposition techniques such as the discrete wavelet transform^15^, empirical mode decomposition^16^, variational mode extraction^18^, and variable-frequency complex demodulation^29^ have also been used for this task. These methods require either the definition of certain frequency bands or the estimation of central frequencies of the respiration which is often impractical, for instance, in pathological breathing (e.g. sleep apnea)^30^ or during periods of stress or relaxation, where subjects can breathe at frequencies outside the standardized bands^31^. Modeling approaches based on Gaussian process assumptions and phase space reconstruction have been recently proposed in^32^. These approaches either assume statistical independence between the ECG components (in the case of the Gaussian process), or require the definition of a time delay and an embedding dimension (in the case of the phase space reconstruction). Other methods extract the respiratory information from the heart rate variability (HRV) signal^33^. Such algorithms are based on the high frequency component of the HRV signal, which is known to be affected not only by respiration but also other factors such as age, physical activity, stress level, and hormones^31^. In addition, they assume that the respiratory modulation of HRV is always present and often constant, which is not always the case. The modulation can sometimes overlap with either sympathetic or other vagal influences unrelated to respiration^8,34^.

Different studies have compared the performance of EDR-based respiratory rate estimation under different conditions, see e.g.^13,27,28^. However, little attention has been paid to the estimation of wave morphology and cardiorespiratory parameters^5,6,11,12,23,28,35^. Information on wave morphology can be used to detect breathing phases, which in turn can be used for the estimation of tidal volume during exercise^35^. Breathing phases and breathing patterns have also been identified as important biomarkers in heart failure^36^, schizophrenia^28^, and sleep apnea^5,6,37,38^. Additionally, wave morphology is required for determining cardiorespiratory phase synchronization and time delay stability^9,39^. These forms of coupling, together with the respiratory sinus arrythmia, are used to quantify the interactions between respiration and heart rate variability^7–9^.

Few studies have compared different EDR methods for the detection of sleep apnea. In^38^, three EDR methods were compared and used to extract time and frequency domain parameters. In^40^, two EDR methods were compared under a controlled experiment consisting of different postural positions, and during sleep apnea, and in^41^ the QRS area was studied for sleep apnea combined with respiratory myogram interference. These studies concluded that high linear correlation exists between the respiratory effort, recorded around the chest, and the EDR signal extracted from the R-wave amplitude.

The present work evaluates 10 different EDR methods presented in literature, operating in different recording settings and different physiological conditions for the estimation of respiratory rate, respiratory wave morphology, and cardiorespiratory interactions. The methods are suitable for use in single-lead ECG applications, and their computational complexity is low. The performance is investigated in the presence of noise, nonstationarities (e.g. while speaking), baseline wander, normal and pathological breathing, and changes in respiratory rate. Three datasets recorded under different circumstances were used, namely in an ambulatory setting, an experimental setting with relaxed conditions, and a hospital environment.

The remainder of this paper is organized as follows. Section 1 describes the datasets, the EDR methods, and the performance measures. Section 2 presents and compares the results for each EDR and each dataset. The results are then discussed in Section 3 and conclusions are presented in Section 4.

## Materials and Methods

### Datasets

The EDR signals under investigation were obtained from three different datasets: two publicly available in Physionet^42^ and one collected in the sleep laboratory of the Universitaire Ziekenhuizen Leuven, UZ Leuven, Belgium, as part of the OSA+ project.

#### Drivers dataset

This dataset, officially called Stress Recognition in Automobile Drivers, was recorded from 16 healthy volunteers while driving a car in Boston, Massachusetts, USA^43^. Single-lead ECG (lead II) and respiratory effort around the thorax were recorded with sampling rates of 486 Hz and 31 Hz, respectively. The duration ranged between 53 and 92 min (77 ± 11 min). During the first and last 15 min of each recording, the subjects were asked to close their eyes and relax with the car in idle. After the first set of 15 min, the subjects drove through quiet and busy streets for about 25 to 60 min.

#### Fantasia dataset

This dataset consists of ECG and respiratory effort signals collected from 40 healthy volunteers at rest, while watching the movie Fantasia (Disney, 1940)^44^. Volunteers belonged to two groups: 20 young subjects aged between 21 and 34 years, and 20 elderly subjects aged between 68 and 85 years. Single-lead ECG (lead II) and respiratory effort around the thorax were recorded with a sampling rate of 250 Hz. The duration of the recordings ranged from 66 to 156 min (118 ± 11 min).

#### Sleep dataset

Single-lead ECG (lead II) and three different respiratory signals were recorded from 100 patients undergoing polysomnography (PSG). The respiratory signals measure respiratory effort around the thorax and abdomen, recorded using respiratory inductance plethysmography. The nasal airflow was recorded using a pressure sensor. Respiratory and ECG signals were both sampled at a rate of 500 Hz and their duration ranged from 260 to 690 min (541 ± 56 min). Data acquisition was carried out in accordance with the recommendations of the UZ Leuven, Commissie Medische Ethiek. The protocol was approved by the Commissie Medische Ethiek UZ Leuven (ML7962). All subjects gave written informed consent in accordance with the Declaration of Helsinki.

All patients suffered from moderate to severe sleep apnea-hypopnea syndrome, with an apnea-hypopnea index (AHI) larger than 15. The sleep apnea events were annotated by sleep specialists at the UZ Leuven using the AASM 2012 scoring rules^30^. The annotations define relative and absolute time of an event, its duration, and type of respiratory event, i.e., obstructive apnea (OSA); central apnea (CEN); obstructive hypopnea (OSH); hypopnea (HPA); and mixed apnea (MIX).

### Pre-processing

#### ECG

The ECG signals were first normalized by subtracting the mean and dividing by the standard deviation (i.e., the standard score) and then segmented into minutes, with the segments indexed by *k*, with *k* = 1, …, *K* and *K* the total number of signal segments. In total, 1218, 4711, and 53553 min were collected for the Drivers, Fantasia, and Sleep datasets, respectively. Then, a signal quality index (SQI), denoted *q*(*k*), was computed to quantify the presence of artifacts and noise, using the algorithm proposed in^45^. The SQI ranges from 0 to 100, where higher values correspond to better signal quality. Segments with *q*(*k*) > 80 are considered of high quality. Baseline wander was removed from the ECG using a forward/backward, fourth-order Butterworth highpass filter with cutoff frequency at 0.5 Hz. The reason for using this filter relies on the results presented in^46^, where it was identified as one of the most accurate methods and yet simple to implement.

R-peaks were detected using the algorithm described in^47^. Missing beats and false alarms were corrected using the RR interval adjustment algorithm described in^5^. The corrected RR intervals were then used to construct the tachogram, resampled to 5 Hz using cubic spline interpolation.

**Figure 1:**
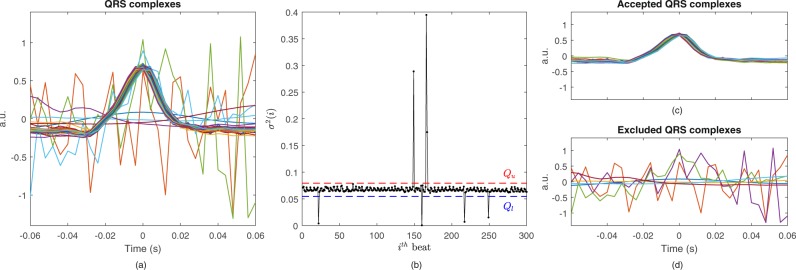
Identification of erroneously detected and abnormal QRS complexes. (**a**) QRS complexes centered around the R-wave. (**b**) QRS variance and the upper (*Q*_*u*_) and lower (*Q*_*l*_) acceptance limits indicated by the dashed lines. Complexes with variance outside these limits were removed from the analysis (**c**). a.u. stands for arbitrary units.

Since the performance of the EDR methods depends on accurate QRS detection, a procedure was implemented to automatically identify aberrant QRS morphologies that might have remained after the RR interval correction. These undesired complexes might still be present in the data due to abnormal or aberrant morphology not detected by the RR interval adjustment, which focuses on rhythm abnormalities. First, each QRS complex was segmented using a window of 60 ms before and after the R-peak, see Fig. 1(a). Then, the mean of each QRS complex was subtracted and its variance computed. This resulted in a time series {*σ*^2^(1), *σ*^2^(2), …, *σ*^2^(*L*)} (Fig. 1(b)), where *L* is the number of QRS complexes in the segment. The 25th (*Q*_1_) and 75th (*Q*_3_) percentiles and the interquartile range (*IQR*) were then computed and the lower (*Q*_*l*_) and upper (*Q*_*u*_) limits of accepted QRS variance were defined as *Q*_*l*_ = *Q*_1_ − 2.5 ⋅ *IQR* and *Q*_*u*_ = *Q*_3_ + 2.5 ⋅ *IQR*. Only QRS complexes fulfilling *Q*_*l*_ < *σ*^2^(*i*) < *Q*_*u*_ were accepted for further analysis. Figures 1(c,d) illustrate QRS complexes accepted and excluded from further analysis, respectively. Note that this procedure is specifically developed for ensembles with beats of the same morphology, where noisy or abnormal beats are in minority. This means that arrhythmias such as bigeminy will not be handled by this procedure.

#### Respiration

**Figure 2:**
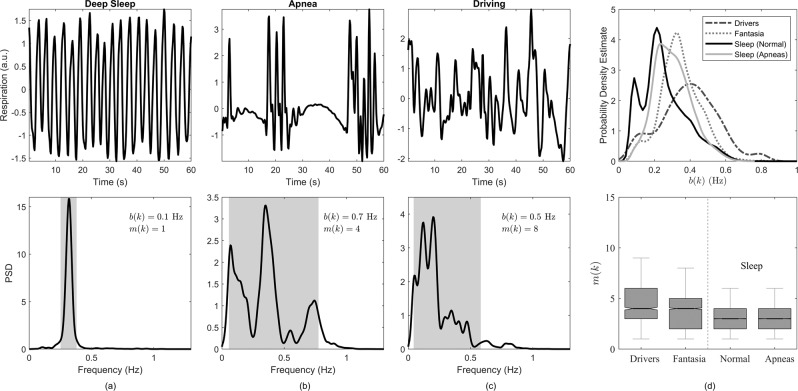
Examples of respiratory segments with different spectral characteristics during deep sleep (**a**), apnea (**b**), and driving (**c**). The PSD of each segment is displayed at the bottom, and the shaded area indicate the bandwidth *b*(*k*). The number of modes *m*(*k*) is also indicated. a.u. stands for arbitrary units. The distribution of *b*(*k*) (top) and *m*(*k*) (bottom) for all datasets are indicated in (**d**).

All respiratory signals were segmented into minutes and downsampled to 5 Hz following antialiasing filtering. Then, forward/backward filtering using a fourth-order Butterworth bandpass filter with cutoff frequencies at 0.05 Hz and 1 Hz was applied. The spectral properties of each segment were used to determine whether the estimation of the respiratory signal and derived parameters deteriorates with more complex respiratory patterns. Therefore, the estimation errors obtained for respiratory signals with a single fundamental frequency were compared to those obtained for more irregular and abnormal respiratory patterns. The spectral properties were characterized using two indices. The first index describes the bandwidth of the respiratory signal, calculated as the width of the frequency band containing 90% of the total power. The power spectral density (PSD) was obtained using Welch’s method with a Hamming window of 30 s, an overlap of 20 s, and 1024 points. The bandwidth, denoted *b*(*k*), is used to differentiate narrow- and broadband respiratory signals. For instance, respiratory signals during periods of relaxation or deep sleep (without the presence of apneas) are characterized by narrow PSDs with a clear respiratory rate, see Fig. 2, where segments during (a) deep sleep, (b) apnea, and (c) driving are exemplified. The segments in Fig. 2(b,c) are characterized by broadband spectra, either due to the presence of artifacts (e.g. during driving) or physiological events like apnea.

The second index is given by the number of modes (i.e. local maxima), denoted *m*(*k*), in the PSD within *b*(*k*). As shown in Fig. 2, *b*(*k*) is larger during both apnea and driving than during deep sleep, however, these patterns can be further differentiated by *m*(*k*). Therefore, both *b*(*k*) and *m*(*k*) are proposed as indicators of respiratory patterns, used for splitting the respiratory signals according to their spectral properties. Figure 2(d) shows the distribution of these indices for the different datasets.

Following pre-processing, each 1-min segment of data consisted of:

- ECG signal
- RR interval tachogram
- Respiratory effort around thorax, denoted ${r}_{th}^{(k)}(n)$, with *n* = 1, …, *N* and *N* the length of the segment, where *N* = 300 for a sampling frequency of 5 Hz.
- Respiratory effort around abdomen, denoted ${r}_{ab}^{(k)}(n)$ — only computed for the Sleep dataset
- Nasal airflow, denoted ${r}_{na}^{(k)}(n)$ — only computed for the Sleep dataset
- ECG signal quality, *q*(*k*)
- Bandwidth of the reference respiratory signal, *b*(*k*)
- Modes in the PSD of the reference respiratory signal, *m*(*k*)

### EDR methods

The following 10 EDR signals are studied, obtained from the accepted QRS complexes indexed by *i*:

*R-wave amplitude* (${\widehat{r}}_{r}(i)$) is simply defined by the R-wave amplitude.

*R-to-S-wave* (${\widehat{r}}_{rs}(i)$) is defined by the difference between the R- and the S-wave amplitudes. The latter is calculated as the minimum amplitude in a 80-ms window after the R-wave^22^.

*Principal component analysis* (${\widehat{r}}_{p}(i)$) accounts for the global variation in amplitude of the QRS samples^11^. The QRS complex is segmented using a symmetric 120-ms window centered around the R-wave, after which all QRSs are organized as rows in a matrix to which principal component analysis (PCA) is applied. The first principal component of the matrix is used as EDR signal.

*Kernel principal component analysis* (${\widehat{r}}_{k}(i)$) is the kernel version of PCA (kPCA). This method first maps the QRS matrix, computed as for ${\widehat{r}}_{p}(i)$ and contained in the input space, into a higher dimensional space by means of a kernel function. The classical PCA is then performed on the transformed dataset, and the first principal component is mapped back to the input space and taken as the EDR signal^12^.

*Q-R slope* (${\widehat{r}}_{up}(i)$) uses the upward slope of the R-wave as the EDR signal^13^. A straight line is fitted to the samples in an 8-ms window centered around the sample with the steepest upward slope. The slope is then used as the EDR signal.

*R-S slope* (${\widehat{r}}_{dw}(i)$) is identical to that of the Q-R slope except that it uses the window centered around the sample with the steepest downward slope^13^. It is important to keep in mind that ${\widehat{r}}_{rs}(i)$, ${\widehat{r}}_{dw}(i)$, and ${\widehat{r}}_{up}(i)$ strongly depend on the ECG lead analyzed, since QRS complexes with prominent R-waves and clear Q- and S-waves are required.

*R-wave angle* (${\widehat{r}}_{\theta }(i)$) is estimated from the QRS slopes (${\widehat{r}}_{up}(i)$ and ${\widehat{r}}_{dw}(i)$), and taken as the EDR signal^13^.

*QRS slope range* (${\widehat{r}}_{sr}(i)$) is defined by the difference between the maximum and the minimum slopes in the QRS complex^27^, computed from the first derivative in a symmetric window of 100-ms centered around the R-wave.

*Central moment* (${\widehat{r}}_{cm}(i)$) is defined by the 4-th order central moment of the bandpass filtered (0.5–45 Hz) ECG signal in the RS interval^14^.

*QRS area* (${\widehat{r}}_{a}(i)$) is defined by the area of the QRS complex^10^.

The EDR signals, sampled at the R-wave positions, were resampled to 5 Hz using cubic spline interpolation, thereby facilitating the comparison with the reference respiratory signals. The signals were then band-pass filtered in the same way as done for the reference. The resulting EDR signals are denoted ${\widehat{r}}_{edr}(n)$, where the subscript *edr* is given by the signal considered, i.e., *edr* ∈ {*r*, *rs*, *p*, *k*, *up*, *dw*, *θ*, *sr*, *cm*, *a*}.

### Performance measures

#### Respiratory rate

The EDR signals are evaluated with respect to respiratory rate, denoted *f*(*k*), computed using the method described in^13^. The method involves the following three steps: PSD estimation, peak-conditioned averaging, and respiratory rate estimation. A subscript is added to *f*(*k*), either *th*, *ab*, or *na*, depending on the reference respiratory signal considered. The notation ${\widehat{f}}_{edr}(k)$ is used when estimated from an EDR signal, e.g., ${\widehat{f}}_{rs}(k)$ indicates that the respiratory rate is estimated from ${\widehat{r}}_{rs}(n)$.

The relative error in the estimation of the respiratory rate, denoted ${e}_{{f}_{edr}}(k)$, is always calculated using ${r}_{th}^{(k)}(n)$ as reference, and is defined by

1 $${e}_{{f}_{edr}}(k)=\frac{| \,{f}_{th}(k)-{\widehat{f}}_{edr}(k)| }{{f}_{th}(k)}\times 100.$$

#### Wave morphology similarity

The similarity between EDR and reference respiratory signals is evaluated using cross-correlation and spectral coherence. In the *k*-th segment, the absolute maximum cross-correlation, denoted ∣*ρ*(*k*)∣, is computed within  ± 3 s. The mean time-frequency (TF) coherence, denoted $\bar{{\gamma }_{r}}(k)$, is computed within the respiratory band *b*(*k*) using the method described in^48^. The mean TF coherence is used to reduce the effect of nonstationarities in each segment.

In all datasets, ${r}_{th}^{(k)}(n)$ is used as reference signal. In the Sleep dataset, two additional reference signals, ${r}_{ab}^{(k)}(n)$ and ${r}_{na}^{(k)}(n)$, are used to evaluate similarity. Thus, ∣*ρ*(*k*)∣ and $\bar{{\gamma }_{r}}(k)$ are also computed between ${r}_{th}^{(k)}(n)$ and ${r}_{ab}^{(k)}(n)$ as well as ${r}_{th}^{(k)}(n)$ and ${r}_{na}^{(k)}(n)$.

#### Cardiorespiratory interactions

An important parameter when investigating different diseases and conditions such as stress and sleep disorders is the amount of information transferred from respiration to heart rate^1,6,49,50^. For example, studies have shown that during apnea the respiratory modulation of the HRV, known as respiratory sinus arrhythmia, is attenuated^4,6,51^. With this in mind, the goal is to determine if the EDR can be used to identify attenuation in cardiorespiratory interactions.

This information is quantified not only for the reference respiratory signals but also for the EDR signals using two novel approaches; the partial TF method^52^ and the transfer entropy obtained using the information decomposition method^49^. The reason for selecting these two approaches relies on the fact that partial TF deals with nonstationarities in the signals and quantifies the coherence between the signals, while transfer entropy explores causality and predictability of the tachogram using the respiratory signal as an independent variable.

##### Partial time-frequency

This method analyzes and interprets systems characterized by two single-inputs and a single-output under nonstationary conditions, using a non-parametric, multivariate quadratic TF representation proposed in^48^. It uses the TF coherence function to decompose the spectrum of the single-output into two spectra reflecting the contributions of the two single, uncorrelated inputs. This method was used in^52^ to quantify the influence of heart rate variability (HRV) on QT interval variability, but here to quantify the cardiorespiratory coupling as the contribution of respiration to the TF spectrum of the tachogram. This contribution, denoted $\bar{{\gamma }_{xy}}(k)$, is quantified by the mean TF coherence between the tachogram and the respiratory signal of the *k*-th segment in the signal. Details on this approach and its implementation can be found in^52^.

The relative error in the estimation of the mean TF coherence is calculated using ${r}_{th}^{(k)}(n)$ as reference, and is defined by

2 $${e}_{{\gamma }_{edr}}(k)=\frac{| {\bar{{\gamma }_{xy}}}_{th}(k)-{\bar{{\gamma }_{xy}}}_{edr}(k)| }{{\bar{{\gamma }_{xy}}}_{th}(k)}\times 100.$$

#### Transfer Entropy

Transfer entropy was computed using information dynamics, being a framework derived from the field of dynamical information theory. Using information dynamics, the amount of information stored in a system and the information transferred from one system to another (i.e. respiration to heart rate) is estimated. In this work, the focus is on the information transferred from **x** to **y**, referred to as transfer entropy *T*_**x**→**y**_; **x** is the respiratory signal, and **y** the tachogram. The larger the amount of information transferred from respiration to heart rate, the larger is the transfer entropy.

A method to quantify *T*_**x**→**y**_ in cardiorespiratory analysis was proposed in^49^. This method links information theory and predictability, resting on the assumption that **x** and **y** are jointly Gaussian. With this assumption, it is possible to describe their dynamics using a linear vector, autoregressive model of order *p*, determined using the Akaike information criterion. In this way, *T*_**x**→**y**_ can be linked to the error probabilities of an autoregressive model, with heart rate and respiration as the dependent and independent variables, respectively.

The relative error in the estimation of the transfer entropy of the *k*-th segment, is calculated using ${r}_{th}^{(k)}(n)$ as reference, and is defined by

3 $${e}_{{T}_{edr}}(k)=\frac{| {T}_{th\to {\bf{y}}}(k)-{T}_{edr\to {\bf{y}}}(k)| }{{T}_{th\to {\bf{y}}}(k)}\times 100.$$

### Statistical analysis

The performance is evaluated at different noise levels, quantified by *q*(*k*). This separation shows the effect of noise on EDR signal morphology and cardiorespiratory parameters, being evaluated using the Kruskal–Wallis test with *α* = 0.05. A multicomparison test with Bonferroni correction was used whenever required.

Using the Sleep dataset, similarity and relative errors are evaluated for normal activity and apnea events. Again, the Kruskal–Wallis test is used with *α* = 0.05 and a multicomparison test with Bonferroni correction.

The relationships between, on the one hand, the similarity and the relative errors *e*_*f*_(*k*), *e*_*γ*_(*k*), and *e*_*T*_(*k*), and, on the other hand, the spectral characteristics *b*(*k*) and *m*(*k*) of the respiration, are evaluated using both Pearson’s and Spearman’s correlation coefficients.

## Results

**Figure 3:**
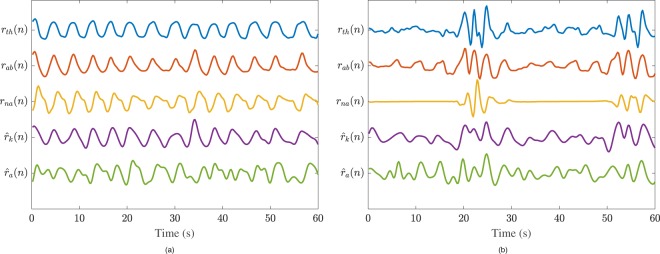
Examples of the reference respiratory and EDR signals computed from two high-quality (*q*(*k*) = 100) segments of the Sleep dataset. Only the EDR signals with the best and worst wave morphology approximations, for these examples, are shown. (**a**) Segment during deep sleep with *b*(*k*) = 0.08 Hz and *m*(*k*) = 1. Correlation and coherence were highest for ${\widehat{r}}_{k}(n)$, i.e., ∣*ρ*∣ = 0.88 and $\bar{{\gamma }_{r}}=0.98$, and worst for ${\widehat{r}}_{a}(n)$, i.e., ∣*ρ*∣ = 0.60 and $\bar{{\gamma }_{r}}=0.82$. (**b**) Segment with an OSA event with *b*(*k*) = 0.57 Hz and *m*(*k*) = 5. Correlation and coherence were highest for ${\widehat{r}}_{k}(n)$, i.e., ∣*ρ*∣ = 0.66 and $\bar{{\gamma }_{r}}=0.79$, and worst for ${\widehat{r}}_{a}(n)$, i.e., ∣*ρ*∣ = 0.47 and $\bar{{\gamma }_{r}}=0.45$. The signal ${\widehat{r}}_{k}(n)$ was inverted to facilitate visualization.

Figure 3 shows two examples of respiratory signals of high-quality ECG segments of the Sleep dataset. These examples illustrate the difference in bandwidth of the reference respiratory signal, *r*_*th*_(*n*) and modes of the respiratory spectrum, quantified by *b*(*k*) and *m*(*k*).

To evaluate the performance, *b*(*k*) was divided into ranges to differentiate between narrow- and broadband respiratory spectra. Table 1 presents the percentage of segments per dataset belonging to each range. Most segments are contained in 0.1 Hz  < *b*(*k*) ≤ 0.5 Hz. About 36% of the segments in the Sleep dataset have a *b*(*k*) ≤ 0.2 Hz, which was expected since more regular respiration is typical during sleep. This is also observed in Fig. 2. Moreover, for the Drivers dataset, 21.7% of the segments are characterized by a bandwidth larger than 0.5 Hz, probably explained by poor-quality recordings or drivers constantly moving and speaking while driving^43^. This observation is supported by the result that the bandwidth was on average 0.26 Hz during the first 15 min when the drivers were relaxed with the car in idle, while the bandwidth increased to 0.45 Hz during driving.

**Table 1:** Bandwidth of segmented respiratory signals.

Bandwidth (Hz)	Number of Segments (%)		
	Drivers	Fantasia	Sleep
*b*(*k*) ≤ 0.1	4.6	1.4	9.1
0.1 < *b*(*k*) ≤ 0.2	9.9	7.3	27.1
0.2 < *b*(*k*) ≤ 0.3	14.3	26.8	44.7
0.3 < *b*(*k*) ≤ 0.4	26	38.8	13.7
0.4 < *b*(*k*) ≤ 0.5	23.5	18.6	3.9
*b*(*k*) > 0.5	21.7	7.1	1.5

Concerning *m*(*k*), there was a significant difference between all datasets, being on average, 4.29 ± 2.10, 3.73 ± 1.71, and 2.93 ± 1.48 for the Drivers, Fantasia, and Sleep datasets, respectively.

The relationship between *b*(*k*) and *m*(*k*) of the respiratory signals and the five performance measures will be presented at the end of this section. First, the performance measures will be discussed.

The ECG signal quality was assessed for all three datasets, resulting in that 2.4%, 3.1%, and 6.1% of the segments were identified as low-quality in the Drivers, Fantasia, and Sleep datasets, respectively. The number of aberrant QRS complexes per segment were different between low- and high-quality segments in all datasets. On average, segments with *q*(*k*) ≤ 80 had 7 ( ≈ 10% per segment) QRS complexes removed from the analysis, while high-quality segments had on average 2 ( ≈ 3% per segment) aberrant QRS complexes. An important observation is that the distributions of the number of segments removed from low- and high-quality segments were right-skewed, with skewness equal to 2.5 and 4.1, and median values of 3 ( ≈ 7% per segment) and 0, respectively. This suggests that *q*(*k*) captures changes in the ECG morphology that could be related to noise and artifacts, and that the excluded QRS complexes in the high quality segments are in minority.

One of the reasons to include the apnea dataset was to evaluate if abrupt changes in the respiratory pattern, like those observed during apneas, affect the estimation of the respiratory parameters. This can be related to either the fact that the morphology of the QRS complexes is affected beyond acceptance, or to the low sensitivity of the EDR to capture those changes. In order to study this, the number of QRS complexes removed from segments containing apneas was compared to the number removed from normal segments (see Table 2).

**Table 2:** Mean and standard deviation of the performance measures for normal and apnea events.

	Type of event					
	Normal	OSA	CEN	OSH	HPA	MIX
*#* aberrant QRS complexes	2 ± 5	2 ± 4	2 ± 3	2 ± 5	2 ± 3	3 ± 3
∣*ρ*∣	0.71 ± 0.2	0.54 ± 0.17^*^	0.58 ± 0.18^*^	0.64 ± 0.18^*^	0.66 ± 0.17^*^	0.45 ± 0.18^*^
$\bar{{\gamma }_{r}}$	0.68 ± 0.24	0.49 ± 0.18^*^	0.55 ± 0.20^*^	0.58 ± 0.20^*^	0.63 ± 0.20^*^	0.39 ± 0.19^*^
*e*_*f*_	3.8 ± 9.4	3.6 ± 8.7	3.2 ± 7.1	4.7 ± 10^*^	3 ± 8.3	3.6 ± 9.4
*e*_*γ*_	23.1 ± 30.8	28.4 ± 34. 2^*^	26.6 ± 30. 6^*^	25.6 ± 32. 2^*^	26.6 ± 30. 4^*^	31.3 ± 32. 9^*^
*e*_*T*_	101 ± 372	109.8 ± 332^*^	80.9 ± 148	115.4 ± 344	83.7 ± 259	76.7 ± 132^*^

Values of ∣*ρ*∣ and $\bar{{\gamma }_{r}}$ were obtained using ${\widehat{r}}_{sr}(n)$, and relative errors were obtained using *r*_*th*_(*n*) as reference and are indicated in %.

^*^Significantly different from normal events.

### Respiratory rate

**Figure 4:**
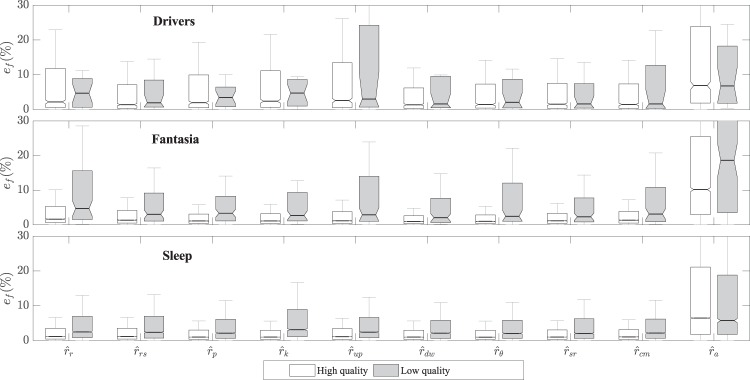
Errors in the estimation of the respiratory rate for high- and low-quality ECG segments.

The estimation errors of the respiratory rate are shown in Fig. 4. The errors associated with the Sleep dataset were significantly lower than those associated with the other two datasets, while the errors associated with the Drivers set were the largest (*p* < 0.05). When looking at each dataset separately, the lowest errors were obtained using ${\widehat{r}}_{rs}(n)$, ${\widehat{r}}_{dw}(n)$, ${\widehat{r}}_{\theta }(n)$, ${\widehat{r}}_{sr}(n)$, and ${\widehat{r}}_{cm}(n)$ for the Drivers dataset, and ${\widehat{r}}_{p}(n)$, ${\widehat{r}}_{k}(n)$, ${\widehat{r}}_{dw}(n)$, ${\widehat{r}}_{\theta }(n)$, and ${\widehat{r}}_{sr}(n)$ for both the Fantasia and Sleep datasets.

In the Sleep dataset, the estimation errors determined during normal activity were compared to those during apneas and presented in Table 2. Only errors obtained for ${\widehat{r}}_{sr}(n)$ are indicated, but they are similar to those obtained for all other EDR signals. In general, the errors were significantly higher (*p* < 0.05) during OSH than during normal activity.

**Figure 5:**
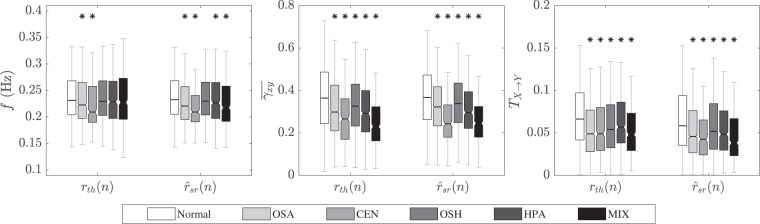
Respiratory rate *f*(*k*) and cardiorespiratory parameters $\bar{{\gamma }_{xy}}(k)$ and *T*_**x**→**y**_(*k*), estimated using *r*_*th*_(*n*) and ${\widehat{r}}_{sr}(n)$ for normal activity and different respiratory events. Significant differences with respect to normal activity are indicated by *.

Regardless of the estimation error observed during OSH and normal activity, the estimated respiratory rates could discriminate (*p* < 0.05) normal activity from OSA, CEN, HPA, and MIX events, see Figure 5 where the results for *r*_*th*_(*n*) and ${\widehat{r}}_{sr}(n)$ are shown. Similar results were obtained for all EDR signals but only those of ${\widehat{r}}_{sr}(n)$ are indicated. The lowest respiratory rates were observed during central events (CEN) for both the reference and estimated respiratory signals.

**Figure 6:**
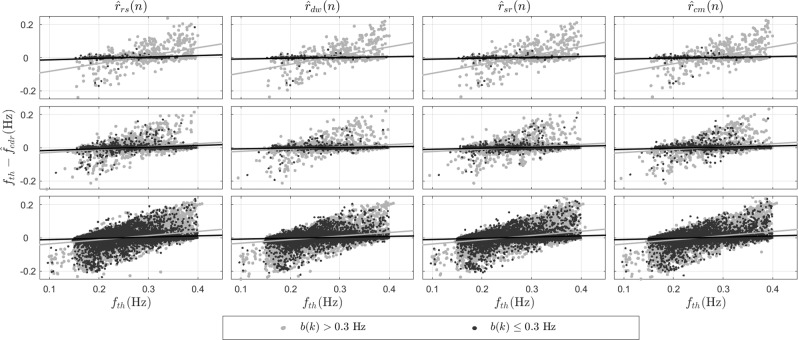
Difference between the reference and estimated respiratory rates, namely ${f}_{th}(k)-{\widehat{f}}_{edr}(k)$, where ${\widehat{f}}_{edr}(k)$ was computed from ${\widehat{r}}_{rs}(n)$, ${\widehat{r}}_{dw}(n)$, ${\widehat{r}}_{sr}(n)$, and ${\widehat{r}}_{cm}(n)$. Differences are given in Hz. Each row corresponds, from top to bottom, to the Drivers, Fantasia, and Sleep datasets. For each case, the least-squares regression line is indicated by a solid line.

The agreement between the reference and estimated respiratory rates was also evaluated at different rates. For illustration purposes, only the results obtained for ${\widehat{r}}_{rs}(n)$, ${\widehat{r}}_{dw}(n)$, ${\widehat{r}}_{sr}(n)$, and ${\widehat{r}}_{cm}(n)$ are indicated in Fig. 6, but the results are similar to those obtained for ${\widehat{r}}_{p}(n)$ and ${\widehat{r}}_{k}(n)$. The least-squares regression line is indicated for each case. For this comparison, a distinction was made between broad- and narrowband respiratory signals using a threshold of *b*(*k*) = 0.3 Hz. Note that for lower rates, the EDR signals tend to overestimate the rate since ${\widehat{f}}_{edr}(k) > {f}_{th}(k)$. The opposite is observed for higher rates. The estimation error, however, is larger at wider bandwidths. The relative errors in the estimation of the respiratory rate were evaluated for different ranges of *f*_*th*_(*k*), and the tendency towards larger errors when *f*_*th*_(*k*) < 0.1 Hz and *f*_*th*_(*k*) > 0.4 Hz was present in all EDRs. These errors were 1.93 ± 0.15% for *f*_*th*_(*k*) < 0.1 Hz, 0.07 ± 0.04% for 0.1 ≤ *f*_*th*_(*k*) < 0.2 Hz, 0.04 ± 0.02% for 0.2 ≤ *f*_*th*_(*k*) < 0.3 Hz, 0.06 ± 0.03% for 0.3 ≤ *f*_*th*_(*k*) < 0.4 Hz, and 0.42 ± 0.07% for *f*_*th*_(*k*) > 0.4 Hz.

### Wave morphology similarity

**Figure 7:**
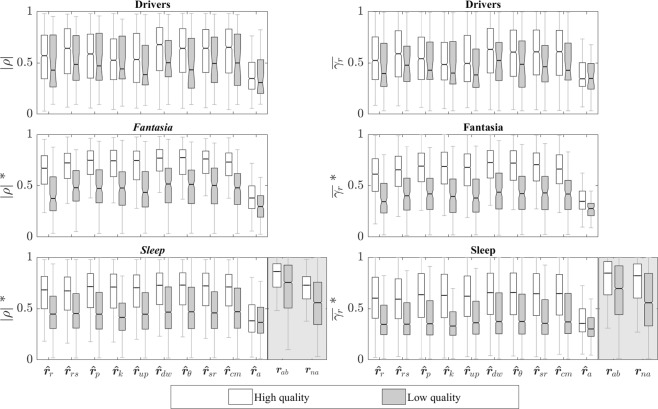
Similarity measures for all datasets and all EDR signals with respect to *r*_*th*_(*n*). The indices *n* were removed to facilitate visualization, i.e., *r*_*th*_(*n*) = ***r***_*th*_. The similarity between *r*_*th*_(*n*) and both *r*_*ab*_(*n*) and *r*_*na*_(*n*) are indicated in the shaded boxes. *Indicate that ∣*ρ*∣ and $\bar{{\gamma }_{r}}$ are significantly different for all EDR signals.

The distribution of ∣*ρ*∣ and $\bar{{\gamma }_{r}}$ for each dataset is shown in Fig. 7, together with those of *r*_*ab*_(*n*) and *r*_*na*_(*n*) for the Sleep dataset. No significant difference between ∣*ρ*∣ and $\bar{{\gamma }_{r}}$ was found in the Drivers dataset, while ∣*ρ*∣ was significantly higher than $\bar{{\gamma }_{r}}$ for all EDR signals in the Fantasia and Sleep datasets.

In the Sleep dataset, the correlation was highest between *r*_*ab*_(*n*) and *r*_*th*_(*n*) since they both correspond to respiratory effort, while the correlation between *r*_*na*_(*n*) and *r*_*th*_(*n*) was comparable to that of the EDR signals. On the other hand, the coherence between the reference respiratory signals was significantly larger than those obtained for the EDR signals. Note that the similarity in the low-quality ECG segments was low also for the reference respiratory signals. This suggests that the SQI actually identifies movement artifacts not only affecting the ECG signals but also the respiratory signals.

A comparison of high- and low-quality segments with *q*(*k*) ≤ 80 was performed for each EDR signal independently. As expected, similarity was lower for low-quality segments, however, the lower similarity was significant (*p* < 0.05) for both the Fantasia and the Sleep datasets, while it was only significant for ${\widehat{r}}_{dw}(n)$, ${\widehat{r}}_{\theta }(n)$, and ${\widehat{r}}_{sr}(n)$ for the Drivers dataset. When comparing datasets, ∣*ρ*∣ and $\bar{{\gamma }_{r}}$ were lowest (*p* < 0.05) for the Drivers dataset for all EDR signals. In contrast, similarity was highest in the Fantasia dataset for all EDR signals, except for ${\widehat{r}}_{r}(n)$ and ${\widehat{r}}_{a}(n)$. The EDR signal most sensitive to noise was ${\widehat{r}}_{k}(n)$, followed by ${\widehat{r}}_{r}(n)$, ${\widehat{r}}_{p}(n)$, and ${\widehat{r}}_{up}(n)$. These EDR signals achieved the largest Spearman correlation (0.25) between both ∣*ρ*(*k*)∣ and $\bar{{\gamma }_{r}}(k)$, and *q*(*k*).

The similarity measures were also analyzed for different ranges of breathing rate. As with respiratory rate estimation, worse wave morphology approximation was obtained for segments with *f*_*th*_(*k*) closer to 0.1 Hz or 0.4 Hz. In these ranges, ∣*ρ*∣ and $\bar{{\gamma }_{r}}$ were lower than 0.3, while for 0.1 ≤ *f*_*th*_(*k*) < 0.4 Hz mean values were always larger than 0.6.

The relationship between the similarity measures and the cutoff frequencies of the high-pass filter used for baseline removal was investigated. These frequencies were 0.1 Hz, 0.3 Hz, 0.5 Hz, and 1 Hz. The original ECG signal and the filtered versions were used to approximate the respiratory wave morphology. The similarity measures were lowest when no baseline wander was removed and highest when the cutoff at 1 Hz was used. It is important to mention that no difference was found between the similarity measures obtained using 0.5 Hz and 1 Hz as cutoff frequencies. For example, ∣*ρ*∣ and $\bar{{\gamma }_{r}}$ values for segments in the Sleep dataset, without baseline removal, were 0.71 ± 0.13 and 0.67 ± 0.16, respectively. After removing the baseline wander using cutoff frequencies of 0.1 Hz, 0.3 Hz, 0.5 Hz, and 1 Hz, ∣*ρ*∣ values were 0.76 ± 0.1, 0.81 ± 0.1, 0.81 ± 0.1, and 0.82 ± 0.1, respectively. Values of $\bar{{\gamma }_{r}}$ were 0.67 ± 0.1 without baseline removal, and 0.70 ± 0.1, 0.74 ± 0.1, 0.76 ± 0.1, and 0.76 ± 0.1, using the aforementioned cutoff frequencies. As can be seen, baseline wander removal improves the results since it enhances the morphological changes modulated by the respiration.

A separate analysis was performed to evaluate if the EDR signals capture ECG baseline information. To this end, the baseline was computed using a cutoff frequency of 0.5 Hz and the EDR signals were computed using the original ECG signals (i.e., before applying the baseline removal step). The similarity measures ∣*ρ*∣ and $\bar{{\gamma }_{r}}$ were then computed between the baseline and the different EDR signals, and values of 0.32 ± 0.15 and 0.37 ± 0.17 were obtained for all EDR signals, respectively. Similar ∣*ρ*∣ and $\bar{{\gamma }_{r}}$ values were obtained between the baseline wander and the reference respiratory signal, namely, 0.34 ± 0.13 and 0.38 ± 0.12, respectively. These results suggest a weak relationship between the baseline wander and the respiratory modulation of the ECG. In other words, the effect of respiration on the ECG signal goes beyond the baseline wander.

In the Sleep dataset, there was a distinction between the similarity during normal activity and apnea events. Correlation and coherence were significantly lower for all EDR signals during OSA and MIX and significantly larger during normal activity (Table 2).

In order to determine if the similarity was affected negatively by the number of excluded QRS complexes per segment, the correlation between this number and the wave morphology similarity was computed for the high-quality segments. Correlation values for all datasets together and all EDR signals were  − 0.04 ± 0.01, confirming that the estimation of wave morphology is not affected by the few ( ≃ 2 for *q*(*k*) > 80) excluded QRS complexes.

After comparing ∣*ρ*∣ and $\bar{{\gamma }_{r}}$ for high-quality segments, the EDR signals which best captured the respiratory changes in time and frequency were ${\widehat{r}}_{rs}(n)$, ${\widehat{r}}_{dw}(n)$, ${\widehat{r}}_{\theta }(n)$, ${\widehat{r}}_{sr}(n)$, and ${\widehat{r}}_{cm}(n)$ for the Drivers dataset, and ${\widehat{r}}_{p}(n)$, ${\widehat{r}}_{k}(n)$, ${\widehat{r}}_{dw}(n)$, ${\widehat{r}}_{\theta }(n)$, ${\widehat{r}}_{sr}(n)$, and ${\widehat{r}}_{cm}(n)$ for both the Fantasia and the Sleep datasets. The signal with the worst performance was ${\widehat{r}}_{a}(n)$, with similarity measures significantly lower than for all other EDR signals in all three datasets.

### Cardiorespiratory interactions

The estimation errors related to cardiorespiratory interaction, quantified using $\bar{{\gamma }_{xy}}(k)$ and *T*_**x**→**y**_(*k*), were evaluated at high signal quality, i.e., *q*(*k*) > 80.

The average estimation error of *T*_**x**→**y**_(*k*) was 50% for all EDR signals and datasets, while the average error of $\bar{{\gamma }_{xy}}(k)$ was 20%. For $\bar{{\gamma }_{xy}}(k)$, the largest errors (*p* < 0.05) in all datasets were obtained for ${\widehat{r}}_{a}(n)$, while the lowest errors were obtained for ${\widehat{r}}_{dw}(n)$ and ${\widehat{r}}_{\theta }(n)$ in the Drivers dataset, ${\widehat{r}}_{rs}(n)$, ${\widehat{r}}_{up}(n)$, ${\widehat{r}}_{dw}(n)$, ${\widehat{r}}_{\theta }(n)$, ${\widehat{r}}_{sr}(n)$, and ${\widehat{r}}_{cm}(n)$ for the Fantasia dataset, and ${\widehat{r}}_{dw}(n)$, ${\widehat{r}}_{\theta }(n)$, and ${\widehat{r}}_{sr}(n)$ for the Sleep dataset. Concerning the estimation of *T*_**x**→**y**_(*k*), the largest errors in all datasets were obtained for ${\widehat{r}}_{cm}(n)$, and the lowest errors were obtained for ${\widehat{r}}_{p}(n)$, ${\widehat{r}}_{k}(n)$, ${\widehat{r}}_{dw}(n)$, ${\widehat{r}}_{\theta }(n)$, and ${\widehat{r}}_{a}(n)$ for the Fantasia dataset, and by ${\widehat{r}}_{sr}(n)$ and ${\widehat{r}}_{a}(n)$ for the Sleep dataset. In the Drivers dataset, all errors were comparable, except those of ${\widehat{r}}_{cm}(n)$, and no significant difference was found between them.

The relative estimation errors in cardiorespiratory interactions were evaluated for different types of apneas and respiratory signals, and the lowest errors were obtained for ${\widehat{r}}_{rs}(n)$, ${\widehat{r}}_{p}(n)$, ${\widehat{r}}_{k}(n)$, ${\widehat{r}}_{dw}(n)$, and ${\widehat{r}}_{sr}(n)$. The errors between the cardiorespiratory parameters estimated using ${\widehat{r}}_{sr}(n)$ and ${\widehat{r}}_{th}(n)$ are presented in Table 2. These errors were similar to those obtained between ${\widehat{r}}_{sr}(n)$ and *r*_*ab*_(*n*). When the nasal airflow was used as reference, the errors almost doubled for the obstructive events, which could be explained by the fact that the respiratory effort continues during obstructive apneas, while the airflow is completely interrupted. The errors for normal and segments with central apneas remained the same.

Both $\bar{{\gamma }_{xy}}(k)$ and *T*_**x**→**y**_(*k*) were able to identify respiratory events (Fig. 5). In other words, their absolute values were significantly larger (*p* < 0.05) for normal activity than for apnea events, capturing “weaker” cardiorespiratory interactions during apnea events. This effect was observed for all EDR signals except for ${\widehat{r}}_{a}(n)$.

**Table 3:** Best performing EDR signals for different experiments and datasets.

	Respiratory Rate						Wave Morphology						Cardiorespiratory Interactions						Rank	
	Drivers		Fantasia		Sleep		Drivers		Fantasia		Sleep		Drivers		Fantasia		Sleep			
	high	low	high	low	high	low	high	low	high	low	high	low	high	low	high	low	high	low	high	low
${\widehat{{\boldsymbol{r}}}}_{r}(n)$																			0	0
${\widehat{{\boldsymbol{r}}}}_{rs}(n)$	X						X								X			x	3	1
${\widehat{{\boldsymbol{r}}}}_{p}(n)$			X		X				X		X								4	0
${\widehat{{\boldsymbol{r}}}}_{k}(n)$			X		X				X		X								4	0
${\widehat{{\boldsymbol{r}}}}_{up}(n)$															X				1	0
${\widehat{{\boldsymbol{r}}}}_{dw}(n)$	X		X		X	x	X		X		X	x	X		X		X	x	**9**	3
${\widehat{{\boldsymbol{r}}}}_{\theta }(n)$	X		X		X	x	X		X		X	x	X		X		X	x	**9**	3
${\widehat{{\boldsymbol{r}}}}_{sr}(n)$	X		X		X	x	X		X		X				X		X	x	8	2
${\widehat{{\boldsymbol{r}}}}_{cm}(n)$	X					x	X		X		X	x			X				5	2
${\widehat{{\boldsymbol{r}}}}_{a}(n)$																			0	0

Results are indicated for the high (*q*(*k*) > 80) and low (*q*(*k*) ≤ 80) quality segments. The marked signals correspond to those with the lowest (*p* < 0.05) errors, additionally, no significant difference was found among those marked. The rank indicates the number of experiments for which the signal performed best.

Table 3 presents the EDR signals that achieved the highest similarity and the lowest estimation errors in the different datasets. The marked signals produced results that were not significantly different (*p* > 0.05) among each other but were significantly different (*p* < 0.05) than those produced by the signals not indicated in the table. Results are indicated for the high- and low-quality segments. In 6 out of the 9 experiments, there was no significant difference between the estimated errors produced by the different EDR signals in the low-quality segments.

### Relationship with the spectral properties of the respiration

The relationship between the five performance measures and the spectral characteristics (*b*(*k*) and *m*(*k*)) of the respiratory signal was evaluated using Pearson’s and Spearman’s correlation coefficients. All results were comparable for both coefficients and for all EDR signals, except for ${\widehat{r}}_{a}(n)$, where correlation values close to 0.1 were achieved for all parameters. For the other 9 EDR signals, the average correlation coefficients between the similarity measures and *b*(*k*) and *m*(*k*) were  −0.5. These results suggest a weak inverse linear relationship between the spectral complexity of the reference signal and its morphologic approximation. Concerning the relationship between the respiratory rate, cardiorespiratory interactions, and *b*(*k*) and *m*(*k*), a weak one was observed with correlation coefficients lower than 0.4.

## Discussion

In total, 10 EDR algorithms were compared in 3 datasets and 3 different tasks (i.e., 9 experiments). The EDR signals that performed best in at least 5 experiments and in high quality segments were ${\widehat{r}}_{dw}(n)$, ${\widehat{r}}_{\theta }(n)$, ${\widehat{r}}_{sr}(n)$, and ${\widehat{r}}_{cm}(n)$ (Table 3). On the other hand, ${\widehat{r}}_{a}(n)$ produced the worst results in all experiments, whereas ${\widehat{r}}_{r}(n)$, ${\widehat{r}}_{rs}(n)$, ${\widehat{r}}_{p}(n)$, ${\widehat{r}}_{k}(n)$, and ${\widehat{r}}_{up}(n)$ achieved intermediate performance in different experiments but were outperformed by the 4 best EDR signals earlier mentioned. Even though those 4 signals were the most consistent with respect to the approximation of wave morphology, respiratory rate, and cardiorespiratory interactions, there are some considerations that need to be taken into account when selecting an EDR signal for a particular task, namely the expected level of noise and the type of respiratory dynamics.

Concerning the estimation of respiratory rate, ${\widehat{r}}_{dw}(n)$, ${\widehat{r}}_{\theta }(n)$, and ${\widehat{r}}_{sr}(n)$ consistently produced the lowest errors for all 3 datasets. The estimated errors were smaller when the respiratory rate was between 0.1 Hz and 0.4 Hz. This is in agreement with previous studies that reported that the estimation of respiratory rate works better within this range^53,54^. The errors also increased with bandwidth and modes of the power spectrum of the reference respiratory signal (Fig. 6), but were the lowest when compared against the other EDR signals. For example, ${\widehat{r}}_{rs}(n)$ and ${\widehat{r}}_{sr}(n)$ were found to be more robust than ${\widehat{r}}_{r}(n)$, as shown in^55^, and than ${\widehat{r}}_{p}(n)$ and ${\widehat{r}}_{k}(n)$ when broadening the respiratory bandwidth, as shown in this study. This represents a disadvantage of PCA methods since they are not only sensitive to outliers but also to the complexity of the reference respiratory signal. The errors were largest in the Drivers dataset and lowest in the Sleep dataset, with intermediate values for the Fantasia dataset. Although subjects in the Fantasia dataset were "at rest" in a supine position, they were still awake. This means that their respiratory patterns were more complex due to various respiratory and cardiac modulators that act during wake periods^4^. This was confirmed by the spectral parameters, namely *b*(*k*) and *m*(*k*), which were larger in the Fantasia dataset than during sleep (see Fig. 2(d)). Furthermore, a weak inverse relationship was found between these parameters and the estimation errors, which could explain the tendency to larger errors in the Fantasia dataset.

The lowest correlation and coherence were observed in the Drivers dataset, containing generally higher noise levels. Even though subjects had some minutes of relaxation before driving, these periods were not completely stress-free. Subjects were still moving around and some discomfort was observed during the recording of the signals^43^. As a result, the morphology of the ECG is affected not only by respiratory modulation but also by movement, thereby reducing the performance of the EDR signals.

The SQI of the Sleep dataset was significantly lower than that of the Fantasia dataset although larger than for the Drivers dataset. This could be explained by the effect apneas have on the morphology of both ECG and respiratory signals^6^. Despite these morphological ECG changes, the proposed procedure to identify aberrant QRS complexes was able to detect these changes as normal since no difference between the number of excluded complexes was found between normal and apnea segments.

When combining all datasets, the strongest relationship between similarity and signal quality was observed for ${\widehat{r}}_{r}(n)$, ${\widehat{r}}_{p}(n)$, ${\widehat{r}}_{k}(n)$, and ${\widehat{r}}_{up}(n)$, suggesting that these algorithms are the most sensitive to poor signal quality. The results obtained in the Fantasia dataset were compared to those reported in^32^, where two models were used to extract the EDR on the same dataset. One model was based on Gaussian processes (GP) and another on phase space reconstruction (PSR). The same parameter ∣*ρ*∣ was used in^32^ to evaluate performance and mean values of 0.703 and 0.643 were obtained for all 40 subjects using the GP and PSR, respectively. In this study, similar values of ∣*ρ*∣ were obtained for ${\widehat{r}}_{dw}(n)$, ${\widehat{r}}_{\theta }(n)$, and ${\widehat{r}}_{sr}(n)$, namely, 0.717, 0.708, and 0.708, respectively. The advantage of the methods evaluated in this work is that they do not assume independence as in GP and do not require the definition of an embedding as in PSR.

The similarity measures were also computed between the respiratory effort measured around the thorax and the other two respiratory signals available in the Sleep dataset, namely, the respiratory effort around the abdomen and the nasal airflow. As expected, the correlation between the two effort signals were highest, while the correlation between *r*_*th*_(*n*) and *r*_*na*_(*n*) was comparable to the ones obtained between *r*_*th*_(*n*) and most of the EDR signals. This can be explained by the fact that the effort might still be present during obstructive events, whereas the airflow is completely interrupted. Spectral coherence demonstrated that the different EDR signals achieved results comparable to those obtained with the reference respiratory signals, suggesting that spectral information related to respiratory effort can be accurately extracted from the morphological changes of the ECG^10^. If the airflow needs to be analyzed, as typically done in sleep diagnosis, the use of EDR signals may not necessarily be the best option.

After analyzing the correlation and coherence between the reference respiration and the different EDR signals, it was found that the best results were obtained for ${\widehat{r}}_{dw}(n)$, ${\widehat{r}}_{\theta }(n)$, ${\widehat{r}}_{sr}(n)$, and ${\widehat{r}}_{cm}(n)$, and the worst results were produced by ${\widehat{r}}_{a}(n)$. The signals with the best performance under higher noise levels (i.e. in the Drivers dataset) were ${\widehat{r}}_{rs}(n)$, ${\widehat{r}}_{dw}(n)$, ${\widehat{r}}_{\theta }(n)$, ${\widehat{r}}_{sr}(n)$, and ${\widehat{r}}_{cm}(n)$. These signals are simple as they are based on amplitudes and slopes around the R-waves, offering an advantage over the other techniques. Signals based on PCA, i.e., ${\widehat{r}}_{p}(n)$ and ${\widehat{r}}_{k}(n)$, on the other hand, tend to outperform other simpler techniques during quasi-stationary conditions^12^. However, these methods require eigendecomposition of a (kernel) matrix which is computationally demanding. Moreover, PCA-based methods are extremely sensitive to outliers^56,57^ as demonstrated by the poor performance in the Drivers dataset (Table 3). It is well-known that the classical PCA solution is optimal from a least-squares perspective, but also known to be very sensitive to outliers of low signal-to-noise ratio. Therefore, future work could focus on the improvement of PCA-based algorithms by including weighting schemes^58^ or probabilistic approaches^59^.

When comparing the performance of PCA and kPCA, it is clear that there is no benefit in using the latter. The potential advantage of kPCA was not observed on any of the 3 datasets since its performance was very similar to its linear counterpart. This raises the question whether the effect of respiration on ECG morphology is linear and nonlinearities are negligible. Nevertheless, this result could be also caused by the wrong model estimation in kPCA. Future studies should address this problem.

In addition to analyzing respiratory rate and wave morphology, the quantification of cardiorespiratory interactions, using the tachogram and the EDR signals, was evaluated. These interactions were quantified by the coherence between the signals, denoted $\bar{{\gamma }_{xy}}(k)$, and the predictability of the tachogram from the EDR, denoted *T*_**x**→**y**_(*k*). The errors obtained in both cases indicate that the coherence between the signals can be estimated with, on average, errors lower than 0.2 times the reference value. On the other hand, the errors were much larger when predictability was estimated. This can be related either to possible delays in the estimation of an EDR signal that might affect the construction of the autoregressive model used in the calculation of *T*_**x**→**y**_(*k*), or to poor morphology approximation.

In general, the errors in the estimation of cardiorespiratory parameters were significantly larger during apnea events than during normal activity for all EDR signals. This can be explained by the fact that during an apnea event, in particular during obstructive events, the respiratory effort might still be present while no air is entering the lungs. As a result, the information captured by the EDR might be unrelated to respiration, thereby causing an overestimation of the cardiorespiratory interactions. In other words, any EDR signal captures the chest movements while the cardiorespiratory information may capture the actual filling of the lungs and its effect on the heart rate.

It is worth noting that the information of different EDR signals can be fused in order to improve the estimation of the respiratory information. Several fusion methodologies have been proposed in the literature, especially for estimating the respiratory rate^60^. Such fusion has been shown to be more effective when combining EDR signals containing complementary (i.e., non-redundant) respiratory information. Future work could focus on evaluating whether the fusion of the best-performing EDR signals in this study could result in an increased performance or if an improvement could be reached by fusing the worst-performing ones.

Finally, this study evaluated performance on three datasets, recorded with different equipment. The Drivers and the Fantasia datasets were collected using “ambulatory” systems, while the Sleep dataset was recorded using polysomnography. This means that ${\widehat{r}}_{dw}(n)$, ${\widehat{r}}_{\theta }(n)$, ${\widehat{r}}_{sr}(n)$, and ${\widehat{r}}_{cm}(n)$ produced the best results not only during different physiological conditions but also for different recording systems. However, before concluding which signals can be used interchangeably the following considerations need to be taken into account. On the one hand, ${\widehat{r}}_{cm}(n)$ requires R- and S-wave delineation, and additional computations to quantify the changes in the morphology of the interval between these two fiducial points by means of the 4th order central moment. On the other hand, ${\widehat{r}}_{dw}(n)$, ${\widehat{r}}_{\theta }(n)$, and ${\widehat{r}}_{sr}(n)$ only require the detection of the R-wave and a definition of a fixed window around it, which makes them computationally simpler than ${\widehat{r}}_{cm}(n)$. Therefore, since QRS detection is much simpler that QRS delineation, ${\widehat{r}}_{dw}(n)$, ${\widehat{r}}_{\theta }(n)$, and ${\widehat{r}}_{sr}(n)$ are selected as the best performing and simplest to compute EDR signals.

## Conclusions

This study showed that the simplest methods for derivation of respiratory information, namely methods exploring either morphological changes in the segment between the R-wave and the S-wave or the slope range of the QRS complex, can be used to accurately estimate the respiratory wave morphology, respiratory rate, and cardiorespiratory information from the ECG. This result is concluded from analyzing different physiological conditions such as periods of relaxation, stress, and both normal and distorted sleep patterns. Furthermore, real life conditions like changes in baseline, transients, artifacts and noise were also considered for evaluation. These findings are crucial for the development of ambulatory systems that can monitor cardiorespiratory parameters using cheap and easy-to-use technology.

## References

[1] Hernando A (2016). Inclusion of respiratory frequency information in heart rate variability analysis for stress assessment. IEEE J. Biomed. Health.

[2] Masaoka Y, Homma I (1997). Anxiety and respiratory patterns: their relationship during mental stress and physical load. Int. J. Psychophysiol..

[3] Dempsey JA, Veasey SC, Morgan BJ, O’Donnell CP (2010). Pathophysiology of sleep apnea. Physiol. Rev..

[4] Varon, C. & Van Huffel, S. Complexity and nonlinearities in cardiorespiratory signals in sleep and sleep apnea. In *Complexity and Nonlinearity in Cardiovascular Signals*, 503–537 (Springer, 2017).

[5] De Chazal P (2003). Automated processing of the single-lead electrocardiogram for the detection of obstructive sleep apnoea. IEEE Trans. Biomed. Eng..

[6] Varon C, Caicedo A, Testelmans D, Buyse B, Van Huffel S (2015). A novel algorithm for the automatic detection of sleep apnea from single-lead ECG. IEEE Trans. Biomed. Eng..

[7] Choi J, Gutierrez-Osuna R (2011). Removal of respiratory influences from heart rate variability in stress monitoring. IEEE Sens. J..

[8] Varon C (2018). Unconstrained estimation of hrv indices after removing respiratory influences from heart rate. IEEE J. Biomed. Health.

[9] Bartsch RP, Schumann AY, Kantelhardt JW, Penzel T, Ivanov PC (2012). Phase transitions in physiologic coupling. Proc. Natl. Acad. Sci..

[10] Moody GB, Mark RG, Zoccola A, Mantero S (1985). Derivation of respiratory signals from multi-lead ECGs. Proc. Comput. Cardiol..

[11] Langley P, Bowers EJ, Murray A (2010). Principal component analysis as a tool for analyzing beat-to-beat changes in ECG features: application to ECG-derived respiration. IEEE Trans. Biomed. Eng..

[12] Widjaja D, Varon C, Dorado A, Suykens JA, Van Huffel S (2012). Application of kernel principal component analysis for single-lead-ECG-derived respiration. IEEE Trans. Biomed. Eng..

[13] Lázaro J (2014). Electrocardiogram derived respiratory rate from QRS slopes and R-wave angle. Ann. Biomed. Eng..

[14] Schmidt M, Krug JW, Schumann A, Bär K-J, Rose G (2015). Estimation of a respiratory signal from a single-lead ECG using the 4th order central moments. CDBME.

[15] Zhao, Y., Zhao, J. & Li, Q. Derivation of respiratory signals from single-lead ECG. In *2008 Int. Seminar on Future BioMedical Information Engineering (FBIE)*, 15–18 (IEEE, 2008).

[16] Campolo, M. *et al*. ECG-derived respiratory signal using empirical mode decomposition. In *IEEE Int. Sym. Medical Measurements and Applications MeMeA*, 399–403 (IEEE, 2011).

[17] Labate D (2013). Empirical mode decomposition vs. wavelet decomposition for the extraction of respiratory signal from single-channel ECG: A comparison. IEEE Sens. J..

[18] Nazari, M. & Sakhaei, S. M. Variational mode extraction: a new efficient method to derive respiratory signals from ECG. *IEEE J. Biomed. Health* **22** (2018). 10.1109/JBHI.2017.2734074 28783649

[19] Leanderson S, Laguna P, Sörnmo L (2003). Estimation of the respiratory frequency using spatial information in the VCG. Med. Eng. Phys..

[20] Bailón R, Sörnmo L, Laguna P (2006). A robust method for ECG-based estimation of the respiratory frequency during stress testing. IEEE Trans. Biomed. Eng..

[21] Pallas-Areny R, Colominas-Balague J, Rosell FJ (1989). The effect of respiration-induced heart movements on the ECG. IEEE Trans. Biomed. Eng..

[22] Pallas-Areny, R. & Canals Riera, F. Recovering the respiratory rhythm out of the ECG. *Med. Biol. Eng. Comput*.**23 (Supplement, Part1)**, 338–339 (1985).

[23] Correa, L. S., Laciar, E., Torres, A. & Jane, R. Performance evaluation of three methods for respiratory signal estimation from the electrocardiogram. In *2008 30th Annual International Conference of the IEEE Engineering in Medicine and Biology Society*, 4760–4763 (IEEE, 2008). 10.1109/IEMBS.2008.4650277 19163780

[24] Sadr, N., Jayawardhana, M. & de Chazal, P. Sleep apnoea diagnosis using respiratory effort-based signals—a comparative study. In *Proc. IEEE Eng. Med. Biol. Soc*., 1551–1554 (IEEE, 2017). 10.1109/EMBC.2017.8037132 29060176

[25] Mendez, M. O. *et al*. Detection of sleep apnea from surface ecg based on features extracted by an autoregressive model. In *2007 29th Annual International Conference of the IEEE Engineering in Medicine and Biology Society*, 6105–6108 (IEEE, 2007). 10.1109/IEMBS.2007.4353742 18003408

[26] Park S-B, Noh Y-S, Park S-J, Yoon H-R (2008). An improved algorithm for respiration signal extraction from electrocardiogram measured by conductive textile electrodes using instantaneous frequency estimation. Medical & biological engineering & computing.

[27] Kontaxis, S. *et al*. ECG-derived respiratory rate in atrial fibrillation. *IEEE Trans. Biomed. Eng. (in early access)*10.1109/TBME.2019.2923587 (2019). 10.1109/TBME.2019.2923587 31226064

[28] Schmidt M, Schumann A, Müller J, Bär K-J, Rose G (2017). ECG derived respiration: comparison of time-domain approaches and application to altered breathing patterns of patients with schizophrenia. Physiol. Meas..

[29] Dash S, Shelley KH, Silverman DG, Chon KH (2010). Estimation of respiratory rate from ECG, photoplethysmogram, and piezoelectric pulse transducer signals: a comparative study of time–frequency methods. IEEE Trans. Biomed. Eng..

[30] Berry RB (2012). Rules for scoring respiratory events in sleep: update of the 2007 AASM manual for the scoring of sleep and associated events: deliberations of the sleep apnea definitions task force of the American Academy of Sleep Medicine. J. Clin. Sleep Med..

[31] Task Force ESC and NASPE (1996). Heart rate variability: standards of measurement, physiological interpretation and clinical use. Circulation.

[32] Janbakhshi P, Shamsollahi MB (2018). Ecg-derived respiration estimation from single-lead ecg using gaussian process and phase space reconstruction methods. Biomedical Signal Processing and Control.

[33] Bailón, R., Sörnmo, L. & Laguna, P. ECG-derived respiratory frequency estimation. In *Advanced methods and tools for ECG data analysis*, 215–244 (Artech House London, 2006).

[34] Leanderson S, Laguna P, Sörnmo L (2003). Estimation of the respiratory frequency using spatial information in the vcg. Medical engineering & physics.

[35] Milagro, J. *et al*. Electrocardiogram-derived tidal volume during treadmill stress test. *IEEE Trans. Biomed. Eng*.10.1109/TBME.2019.2911351 (2019). 10.1109/TBME.2019.2911351 30990416

[36] Garde A (2017). Assessment of respiratory flow cycle morphology in patients with chronic heart failure. Med. Biol. Eng. Comput..

[37] Krieger J, Sforza E, Boudewijns A, Zamagni M, Petiau C (1997). Respiratory effort during obstructive sleep apnea: role of age and sleep state. Chest.

[38] Sadr, N. & de Chazal, P. A comparison of three ECG-derived respiration methods for sleep apnoea detection. *Biomed. Phys. Eng. Express*. (2019).

[39] Bashan A, Bartsch RP, Kantelhardt JW, Havlin S, Ivanov PC (2012). Network physiology reveals relations between network topology and physiological function. Nat. Commun..

[40] O’Brien C, Heneghan C (2007). A comparison of algorithms for estimation of a respiratory signal from the surface electrocardiogram. Comput. Biol. Med..

[41] Maier C, Wenz H, Dickhaus H (2014). Robust detection of sleep apnea from holter ECGs. Method. Inform. Med..

[42] Goldberger A (2000). Physiobank, physiotoolkit, and physionet: Component of a new research resource for complex physiologic signals. Circulation.

[43] Healey JA, Picard RW (2005). Detecting stress during real-world driving tasks using physiological sensors. IEEE Trans. Intell. Transp..

[44] Iyengar N, Peng C, Morin R, Goldberger AL, Lipsitz LA (1996). Age-related alterations in the fractal scaling of cardiac interbeat interval dynamics. Am J Physiol-Reg I.

[45] Moeyersons J (2019). Artefact detection and quality assessment of ambulatory ECG signals. Comput. Methods Programs Biomed..

[46] Lenis, G., Pilia, N., Loewe, A., Schulze, W. H. & Dössel, O. Comparison of baseline wander removal techniques considering the preservation of ST changes in the ischemic ECG: a simulation study. *Comput. Math. Method. M*.**2017** (2017). 10.1155/2017/9295029 PMC5361052 28373893

[47] Moeyersons J, Amoni M, Van Huffel S, Willems R, Varon C (2019). R-deco: An open-source matlab based graphical user interface for the detection and correction of r-peaks. PeerJ Computer Science.

[48] Orini M, Bailon R, Mainardi LT, Laguna P, Flandrin P (2012). Characterization of dynamic interactions between cardiovascular signals by time-frequency coherence. IEEE Trans. Biomed. Eng..

[49] Faes L (2015). Information decomposition in bivariate systems: theory and application to cardiorespiratory dynamics. Entropy.

[50] Kontaxis S, Lázaro J, Gil E, Laguna P, Bailón R (2019). Assessment of quadratic nonlinear cardiorespiratory couplings during tilt-table test by means of real wavelet biphase. IEEE Trans. Biomed. Eng..

[51] Penzel T (2007). Cardiovascular and respiratory dynamics during normal and pathological sleep. Chaos: An Interdisciplinary Journal of Nonlinear Science.

[52] Orini M, Pueyo E, Laguna P, Bailón R (2018). A time-varying nonparametric methodology for assessing changes in QT variability unrelated to heart rate variability. IEEE Trans. Biomed. Eng..

[53] Lázaro J, Nam Y, Gil E, Laguna P, Chon KH (2015). Respiratory rate derived from smartphone-camera-acquired pulse photoplethysmographic signals. Physiological measurement.

[54] Lázaro, J. *et al*. Pilot study on electrocardiogram derived respiratory rate using a wearable armband. In *2018 Computing in Cardiology Conference (CinC)*, vol. 45, 1–4 (IEEE, 2018).

[55] Mason, C. & Tarassenko, L. Quantitative assessment of respiratory derivation algorithms. In *Proc. IEEE Eng. Med. Biol. Soc*., vol. 2, 1998–2001 (IEEE, 2001).

[56] Jolliffe IT, Cadima J (2016). Principal component analysis: a review and recent developments. Phil. Trans. R. Soc. A.

[57] Varon, C. & Van Huffel, S. ECG-derived respiration for ambulatory monitoring. In *Proc. Comput. Cardiol*., 169–172 (IEEE, 2015).

[58] Bailey S (2012). Principal component analysis with noisy and/or missing data. Publications of the Astronomical Society of the Pacific.

[59] Tipping ME, Bishop CM (1999). Probabilistic principal component analysis. Journal of the Royal Statistical Society: Series B (Statistical Methodology).

[60] Charlton PH (2016). An assessment of algorithms to estimate respiratory rate from the electrocardiogram and photoplethysmogram. Physiol. Meas..

